# 

**Published:** 1885-06

**Authors:** 


					?diipoi^ial, ?hg.
American Dental Association.?We direct special
attention to the meeting of this association, to be held in Min-
neapolis, Minn., commencing Tuesday, August 4, 1885, at 11
A. M. From the notice of arrangements and attractive
natural features of the country as portrayed by Dr. Crouse
in the call for this meeting which appeared in the last number
of this Journal, the attendance will no doubt be very large,
and the proceedings of great interest.
To Readers and Correspondents
Communications intended for publication, and exchange journals and
papers should be addressed to the Editor of the American Journal of
Dental Science, 259 N. Eutaw St., Baltimore, Md. All letters relating
to the business of the Journal, or containing cash remittances, to be
directed to Messrs. Snowden & Cowman. Articles for publication should
be received by the editor at least three weeks previous to the first month
on which the number in which it is designed for them to appear, is issued.
The American Journal of Dental Science will contain fifty
pages of reading matter in each number, making a volume of about
Six Hundred pages,
EXCLUSIVE OF ADVERTISEMENTS.

				

